# ISCEV standard for clinical pattern electroretinography (2024 update)

**DOI:** 10.1007/s10633-024-09970-1

**Published:** 2024-03-15

**Authors:** D. A. Thompson, M. Bach, J. J. McAnany, M. Šuštar Habjan, S. Viswanathan, A. G. Robson

**Affiliations:** 1 The Tony Kriss Visual Electrophysiology Unit, Clinical and Academic Department of Ophthalmology, Sight and Sound Centre, Great Ormond Street Hospital for Children NHS Trust, London, WC1N 3AJ UK; 2 Great Ormond Street Institute for Child Health, University College London, London, UK; 3 Eye Center, Medical Center - Faculty of Medicine, University of Freiburg, Freiburg, Germany; 4 Department of Ophthalmology and Visual Sciences, University of Illinois at Chicago, Chicago, IL USA; 5 Department of Bioengineering, University of Illinois at Chicago, Chicago, IL USA; 6 Eye Hospital, University Medical Centre Ljubljana, Grablovičeva ulica 46, 1000 Ljubljana, Slovenia; 7 Department of Biological and Vision Sciences, State University of New York College of Optometry, New York, NY USA; 8 Department of Electrophysiology, Moorfields Eye Hospital, 162 City Road, London, EC1V 2PD UK; 9 Institute of Ophthalmology, University College London, London, UK

**Keywords:** Clinical electrophysiology, Clinical standards, Electroretinogram, International society for clinical electrophysiology of vision (ISCEV), Pattern electroretinogram, PERG, Retina, Retinal ganglion cells, Visual evoked potential

## Abstract

The pattern electroretinogram (PERG) is a localized retinal response evoked by a contrast-reversing pattern, usually a black and white checkerboard, which provides information about macular and retinal ganglion cell function. This document, from the International Society for Clinical Electrophysiology of Vision (ISCEV; www.iscev.org) presents an updated and revised Standard for clinical PERG testing. This replaces the 2013 and all earlier versions. Minimum protocols for basic PERG stimuli, recording methods and reporting are specified, to promote consistency of methods for diagnosis and monitoring purposes, while responding to evolving clinical practices and technology. The main changes in the updated ISCEV Standard for clinical PERG include expanded guidance about large stimulus fields, stimulus parameters for simultaneous PERG and pattern visual evoked potential recording, baseline drift correction, and use of consistent ambient room lighting. These changes aim to provide a clinically relevant document about current practice which will facilitate good quality recordings and inter-laboratory comparisons.

## Introduction

The pattern electroretinogram (PERG) is an established clinical method of assessing macular and retinal ganglion cell (RGC) function. The PERG localizes the retinal response by temporally modulating a patterned stimulus of constant mean luminance. The International Society for Clinical Electrophysiology of Vision (ISCEV) Standard clinical PERG is recorded to an abrupt contrast reversal of equal numbers of black and white checks. Fixation is at the node of the central checks, localizing the stimulus to the retinal area centered on the macula. The PERG is of relatively low amplitude compared with the full-field electroretinogram (ERG) e.g., typically 2 orders of magnitude smaller, and obtaining reliable results requires careful attention to technique, including stimulus and electrode quality as well as to sources of extraneous noise. This document updates the ISCEV Standard for clinical PERG and supersedes the version published in 2013 [1]. The Standard will apply equally to adults and children able to comply with testing.

ISCEV publishes and maintains other standards for clinical electrophysiological tests, often used to complement PERG testing. These include Standards for full-field ERG testing [2], multifocal electroretinography (mfERG) [3], electro-oculography [4] and visual evoked potentials (VEP) [5]. There is also a guideline for calibration and verification of stimuli and recordings instruments for use in clinical electrophysiology [6]; a guide to visual electrodiagnostic procedures which highlights the typical clinical applications of all ISCEV standard tests including the PERG [7]. In addition, extended protocols are published and may be indicated for enhanced or supplementary characterization. These include the dark adapted red flash ERG [8], photopic On–Off ERG [9], S-cone ERG [10], the stimulus response series for light adapted full-field ERG [11] and dark-adapted full field ERG b-wave [12], the photopic negative response (PhNR) of the full field ERG [13] and an extended protocol for derivation and analysis of the strong flash rod-isolated ERG a-wave [14]. In addition, there is an extended protocol for VEP methods of estimation of visual acuity [15]. The ISCEV website should be consulted for current updates (www.ISCEV.org/standards). This document is not a safety standard, and it does not mandate procedures for individual patients or define the qualifications required of those administering or interpreting the tests.

### Summary of changes to the ISCEV Standard PERG

The main revisions in this Standard include the addition of an optional standardized large field PERG, and an option to perform a standardized simultaneous PERG and PVEP recording. There is a refined definition of a stimulus check width as one that must divide evenly into the stimulus field, and the need for consistency of ambient room lighting is emphasized. The effects of baseline drift on PERG P50 and N95 components and the correction of baseline relative to a pre-stimulus interval is illustrated in new figures that also show an example of a large field PERG. The ratio of N95 to P50 amplitude (N95:P50 amplitude) is included as a means of identifying selective or predominant N95 loss. Details of PERG analysis have been added and reporting specifications are highlighted, including the definition of trigger point, and filter characteristics. The acceptable stimulus reversal rate is changed to 4.0 rps ± 1.0 rps. Two non-standard methods are described, including an expanded section on the steady state PERG and a section highlighting complementary use of coarse pattern stimuli. These changes aim to provide a clinically useful document which will facilitate good quality recordings and inter-laboratory comparisons.

### Overview and retinal origins of the transient PERG

The PERG is dominated by the response of the RGCs, “driven” by the cone photoreceptors and corresponding inner retinal circuitry. The waveform of the PERG produced by the contrast reversal of pattern stimuli partly depends on the temporal frequency of the stimulus (transient versus steady-state). The ISCEV Standard PERG is a transient response i.e., a response that is complete before the next contrast reversal. At low temporal frequencies (less than 6 reversals per second; equivalent to 3 Hz), transient PERGs are obtained with distinct PERG P50 and N95 components, of differing diagnostic significance.

**Fig. 1:**
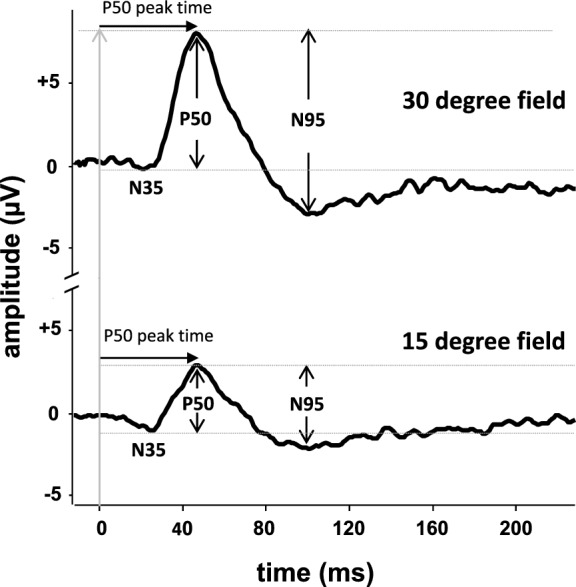
Pattern ERG waveforms. Pattern ERGs recorded to 50’ check widths presented in standard 15-degree and 30-degree fields. P50 and N95 peak to trough amplitude and peak time measures are arrowed. The PERG P50 peak times from the 30- and 15-degree fields are the same, but P50 amplitude is larger from the 30-degree field in a healthy individual

The PERG waveform displays positivity at the active corneal electrode as upward by convention (Fig. 1). In the absence of dysfunction the PERG waveform typically has a small initial negative component with a peak time of approximately 35 ms (N35), followed by a much larger positive component with a peak time in the region of 50 ms (P50). Approximately 70% of P50 is thought to arise in the RGCs cells and 30% from more distal retinal cells including the bipolar cells. The P50 component is followed by a larger negative component with a mean peak time in the region of 95 ms (N95). Both P50 and N95 depend upon the function of the outer retina and macular cones (see Sect. "Clinical applications" for clinical applications below). The RGC contribution to P50 has been suggested to reflect activity close to the ganglion cell bodies, and contributions to N95 more localized to the optic nerve head. It is emphasized that although termed P50 and N95, peak times and amplitudes vary, and reference ranges are essential for meaningful interpretation.

## Clinical applications

Since the PERG (in contrast to the full-field ERG) is a focal response summed from the retinal area covered by the stimulus image, the P50 component can be used as a sensitive indicator of cone system function within the macular region of the retina and P50 reduction and/or delay can characterize macular cone system dysfunction.

The N95 component or N95:P50 amplitude can be used as an indicator of macular RGC function. In severe or chronic retinal ganglion cell dysfunction, there may be P50 reduction, but in such circumstances P50 often shortens in peak time, reflecting loss of the retinal ganglion cell contribution to P50 [7].

The PERG is also influenced by optical factors such as media opacity and refractive error. In the absence of significant optical factors, PERGs have clinical value in both neurological and ophthalmological practice. PERGs can be used in patients with abnormal pattern VEPs to establish if a central retinal disorder is present and thus differentiate between macular cone system dysfunction (maculopathy), RGC or optic nerve pathway dysfunction as a cause for the VEP abnormality. The PERG may also complement the full-field ERG in distinguishing between generalized retinal dysfunction and macular cone dysfunction (or by identifying the severity of macular cone involvement).

The clinical value and application of the PERG and mfERG have some similarities but there are important differences. The ISCEV standard mfERG assesses cone system function over 61 or 103 discrete and small areas of the retina [3], using high photopic luminance and high temporal rates of local luminance change. The PERG is a contrast-dependent response and assesses both macular cone and RGC function, and although both mfERG and PERG require good patient compliance, small fixation errors have a greater impact on the quality of mfERGs due to eccentricity-dependent scaling factors [3] and smaller responses (each derived from a far smaller area than the standard checkerboard). The application of PERG and mfERG in clinical practice, with specific examples including advantages and disadvantages, is further described in the ISCEV guide to visual electrodiagnostic procedures [7].The photopic negative response of the full-field ERG may also be used to assess RGC activity [13] but reflects global function rather than the more localized central retinal area assessed by the PERG, the latter more sensitive to optic nerve disorders that preferentially or selectively involve the papillomacular bundle.

## Technology

Standard equipment for visual stimulus generation, amplification of physiological signals, and the recording and storing of electrophysiologic data is required for PERG testing. Information about the calibration of equipment and measurement of the parameters specified in this standard appears in the ISCEV guideline for calibration and verification of stimuli and recording instruments [6].

### Electrodes

#### Active recording electrodes

Active electrodes connected to the positive input of the recording system should contact the cornea or bulbar conjunctiva and be carefully positioned to optimize response stability. Suitable electrodes include conductive fibres, and conjunctival wire loops. Electrodes that degrade the optical quality of the stimulus on the retina must not be used, including contact lens electrodes. Skin recordings, such as obtained with electrodes on the lower eyelids, are of relatively low amplitude and less suitable for quantification of transient PERGs but may be useful if corneal electrodes are not tolerated or contraindicated. Many more trials may need to be averaged to improve the signal to noise ratio. If skin active electrodes are used this should be documented as a departure from the ISCEV standard.

It is incumbent upon practitioners to master the technical requirements of the electrode type used to obtain good contact and consistent electrode positioning across patient and reference groups. Those who perform PERGs should be aware of possible causes of electrode-associated artefact. Impedance measurements of active electrodes in contact with the tear film may not be necessary as these are generally very low. Any measurements taken must use microcurrents from electrically isolated medical grade impedance meters (see Sect. "Patient isolation").

#### Reference electrodes

Reference electrodes are connected to the negative input of the recording system to enable a potential difference to be recorded. The reference is a skin electrode placed near the ipsilateral orbital rim or outer canthus temporal to each eye. The use of other reference locations, such as the mastoid, earlobe or forehead, can result in intrusion from cortical evoked potentials and should not be used.

#### Common electrode

A separate (ground) electrode is attached to an indifferent point and connected to the common (ground) input of the recording system to achieve common mode rejection of extraneous electrical noise. Typical locations are on the forehead, earlobe or mastoid. The location of the ground electrode does not affect the PERG.

#### Skin electrode characteristics

The impedance of passive skin electrodes measured between 10 and 100 Hz should normally be 5 kΩ or less. For maximal suppression of mains interference the recording and reference electrodes should have similar impedance levels. The skin should be prepared by cleaning, and a suitable conductive paste or gel is applied to ensure good electrical connections.

#### Electrode stability

In the absence of pattern stimulation and eye movement, the baseline voltage should be stable. It is recommended that the raw input trace is monitored. In order to achieve stability, the reference electrodes may need to be non-polarizable, for example AgCl/Cl electrodes.

#### Electrode cleaning

Recording PERGs involves the exposure of corneal electrodes to tears, and there is potential exposure of skin electrodes to blood if there is any break in the surface of the skin. Reusable electrodes must be cleaned and sterilized after each use to prevent transmission of infectious agents. The cleaning protocol should follow the manufacturers’ recommendations and meet current local and national requirements for devices that contact skin and tears.

### Stimuli

#### Field size and check width

The stimulus for the standard PERG is a black and white reversing checkerboard. The width of the individual checks for the standard PERG is 0.8° (± 0.2°) [48 min of arc, range 36’ – 60’ check width], and the checks should be square (± 5% error). It is highlighted that a check size between 0.8 degrees and 1 degree will overlap with a check size range specified in the ISCEV Standard VEP. It is not necessary to use a square stimulus field, but the aspect ratio between the width and the height of the stimulus field should be between 4:3 and 1:1. The mean of the width and the height of the stimulus field should be 15° (± 3°).

It is important to select a check width that divides the field width into an even number of black and white elements. This will ensure there are no local luminance changes with each reversal (see screen luminance below).

Additional use of non-standard larger and smaller checks may have clinical value if appropriate reference ranges are available e.g., the use of large checks can be helpful in some cases of low vision, but these stimuli are not covered by this Standard.

#### Screen luminance

The PERG has lower amplitude with low stimulus luminance. A photopic luminance for the white areas of greater than 80 cd·m^−2^ is required. The mean luminance of the stimulus screen must be constant during checkerboard reversals (i.e., no transient luminance change).

Note: whilst some liquid crystal display (LCD) stimulators can present a brief luminance artefact during pattern reversal, rendering them unsuitable for PERG recording, some manufacturers have reduced this on off timing artefact to acceptably low levels for PERG recording. It is important to check for luminance artefact when assessing screen technology for PERG [6].

#### Contrast

The contrast between black and white squares should be maximal (close to 100%) for the standard PERG and not less than 80%. Low stimulus or retinal contrast diminishes the amplitude of the P50.

#### Background ambient illumination

It is essential to use the same ambient lighting conditions for all PERG recordings. Dim or ordinary room lighting is appropriate and must replicate the conditions used for the collection of reference data. Note: the PERG P50 peak time recorded in bright ambient room light can be shorter (by up to approximately 5 ms) than in a dark room illuminated only by the visual display unit (VDU) stimulus. Care should be taken to keep bright lights out of the subjects’ direct view and away from the VDU where it may produce veiling glare and reduce the stimulus contrast.

#### Reversal rate

The Standard transient PERG should be obtained using a reversal rate 4 ± 1 reversals per second (rps). Reversal rate must be reported in rps, not in Hertz (Hz). It may take up to 200 ms to recover to baseline and rates higher than 5 rps should not be used. Slower rates (1.8–2.2 rps) would comply with current ISCEV VEP standard [5] but are not efficient for recording the PERG. For simultaneous recording of PERG and PVEP see Sect. "Simultaneous PERG and PVEP" (below).

#### Visual display units (VDUs) for stimulation

The technology of VDUs used to present pattern stimuli may affect stimulus definition and timing. The frame rate of the VDU is a significant stimulus parameter for PERGs. For raster-based CRTs, a frequency of 75 Hz or greater should be used. Most current LCDs present a static, non-flickering image, typically refreshed at 60 Hz. Rarely used but possible are projection systems, plasma displays and displays using OLEDs (organic light-emitting diodes) to create the image. LCD-based displays and projectors may have a significant delay between signal input and stimulus output. This delay must be taken into account when defining time zero of the PERG. If separate VDUs are used the VDU manufacturer should provide details about the delay of the display device; otherwise, it is possible to measure the delay with a photodiode.

The frame rate or refresh rate affects PERG peak time, because ‘‘time zero’’ is conventionally defined as the time at which the refresh begins at the top of the screen. It is acknowledged that for PERG it is better to define this value as the time where the center of the screen is updated, but this definition is not widely used and shortens all peak times by at least 5 ms. Manufacturers of integrated VDUs and recording systems should provide both the refresh rate and the screen location that is defined as time zero to allow peak times to be interpreted correctly and compared between laboratories.

### Recording equipment

#### Patient isolation

Electrical isolation of the patient should be ensured according to the current safety standards of the country in which the test is performed. In the absence of national requirements, the equipment should meet a general standard, such as Medical Electrical Equipment IEC 60601–1.

#### Amplification

Amplifiers with a minimum input impedance of 10 MΩ are required. The system should be capable of recording frequencies across a broad range, and a band-pass filter should be applied that includes the minimum frequency range of 1 to 100 Hz (corner frequencies defining 3 dB attenuation). Users should be aware that changes to the filter characteristics or corner frequencies of the high-pass and low-pass filters may change the amplitude and peak times of the PERG. Analogue notch filters (that suppress signals at the mains line frequency) are contraindicated, as they may reduce or distort the signal. Some users may encounter severe electromagnetic interference from the stimulus display that makes it difficult to obtain satisfactory recordings with these filter settings. Ideally, such interference should be eliminated at source by shielding or by modifying the equipment. Rearranging the electrode leads (such as grouping to a point behind the patient’s head) may also be of benefit.

#### Averaging and signal analysis

**Fig. 2:**
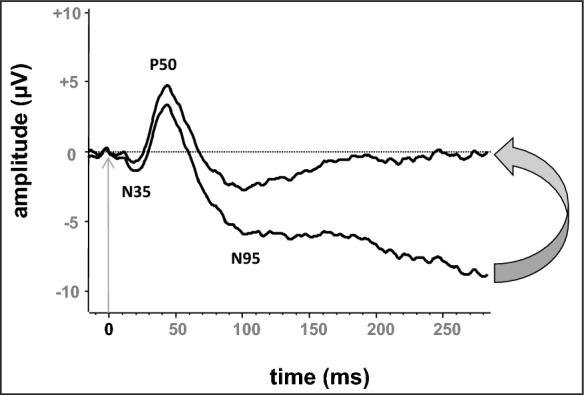
Baseline drift. Pattern ERG showing the effect of baseline drift on P50 and N95 measurements and the ratio of N95 to P50 amplitude, (see Sect. "Averaging and signal analysis" Averaging and signal analysis). The arrow shows the result of removing the linear trend (detrending)

Signal averaging is necessary because of the small amplitude of the PERG. The analysis period (sweep time) for the standard PERGs should be 150 ms or greater as it can take 200 ms for the PERG to recover to baseline. A pre-stimulus baseline of 20 ms or more is recommended to facilitate detection and compensation of any baseline drift which may distort N95 measurements, e.g., using trend removal software during analysis (Fig. 2).

#### Artefact rejection

Computerized artefact rejection is essential. The limits for rejection should be set at no higher than ± 100 µV. Any post hoc manual rejection of individual sweeps confounded by obvious artefacts such as, eye movement and blink which may deflect the pre-baseline or cause the trace to fall out of the response window, should use a consistent criterion that leaves sufficient sweeps for an average.

#### Sampling rate

A minimum sampling rate of 1,000 Hz (1 ms per point) is recommended. See the ISCEV guideline for calibration and verification of stimuli and recording instruments [6] for further information.

#### Data display system

Display systems must have adequate resolution to represent accurately the characteristics of this small amplitude signal. Ideally, the recording system provides simultaneous display of the input signal (i.e., the response generated by each check reversal) and the accumulating average. In the absence of a simultaneous display, a rapid alternation between displaying the input signal and displaying the current average is advisable, so that the quality of the input signal can be adequately monitored. Even with a computerized artefact rejection system, it is important that the input signal be continuously monitored for baseline stability and for the absence of amplifier saturation.

#### Calibration

All stimulus parameters including luminance and contrast should be calibrated either locally or by the manufacturer. Calibration of amplifier gain is assessed by passing a known signal, with amplitude and timing in the range of the physiological signals through the entire system as described elsewhere [6]. Regular recalibration is advised [6].

## Clinical protocol

### Preparation of the patient

#### Positioning, pupils and pre-adaptation

Patients should be as comfortable as possible with their head in a stable position. A headrest may be helpful, but a chinrest is inappropriate because it can increase muscle artefacts. The PERG must be recorded without dilation of the pupils to maximize retinal image quality. In case of extreme pupil size (miosis, mydriasis) or anisocoria, pupil diameters should be documented. A pre-adaptation period is not necessary, but imaging techniques that use strong illumination should be avoided directly before PERG testing. Ocular ultrasound should also be avoided prior to PERG testing, to avoid degradation of optical quality due to ultrasound gel on the ocular surface. In case of dry eye, the use of methylcellulose based artificial tears or buffered saline drops is recommended.

#### Fixation

Stable fixation is essential to obtain reliable PERG recordings and a fixation mark at the node of the central checks of the checkerboard should be provided. If unable to see the fixation mark patients should be directed to look towards the center of the screen and encouraged to keep their eyes still. Cross hairs may be helpful in cases of central scotoma. Fixation should be monitored, preferably using a monitoring camera that allows close visualization of the pupil, or by asking patients to point at the middle of the screen e.g., with a laser pointer throughout the test. Excessive blinking during recording should be discouraged. Instead, pauses in recording may be advantageous to allow gentle blinking. It is useful to instruct the patient to close their eyes every 10 to 15 s and resume the recording after a few seconds of pause when fixation is regained, and the input baseline is stable again. Although controlled blinking with interrupted averaging allows tears to lubricate the corneal surface, additional application of methylcellulose based artificial tears or buffered saline drops may improve patient comfort and compliance. The application of short acting topical anesthesia can be used and, although rarely necessary, can improve comfort and reduce blinking if excessive or problematic.

#### Refraction

Optimal image quality is necessary for PERG recordings because of the sensitivity of P50 amplitude to contrast loss and small amounts of blur. Patients can wear the appropriate optical correction for the test distance, or trial lenses can be used; bifocals or progressive glasses may not ensure optimal imaging over the full stimulus field. The near optimal correction is particularly important for patients with reduced accommodation (such as patients with presbyopia over 50 yr of age), especially for displays that require a short viewing distance to achieve the stimulus field.

To calculate the positive near lens power in diopters, which needs to be added to the distance spectacle prescription for optimal acuity, 100 cm is divided by the stimulus viewing distance (cm). Thus optimal focus at viewing distances of 100 cm require an additional + 1 diopter, at 50 cm + 2 diopters, at 33 cm + 3 diopters, at 25 cm + 4 diopters.

#### Monocular versus binocular recording

Binocular PERG recording is recommended as it reduces examination time and facilitates more stable fixation. In cases of asymmetric visual loss, it allows fixation by the better eye and thus provides a more reliable interocular comparison, since both eyes are recorded under identical recording conditions. Monocular stimulation is required for patients with ocular misalignment (e.g., strabismus) and for recording of the PERG and the monocular VEP simultaneously.

#### Recording

A minimum of 100 artefact-free sweeps should be collected and averaged for a standard PERG. Up to 300 sweeps may be needed when the PERG is small or undetectable, or in conditions with high levels of background noise. Interrupted averaging may prevent fatigue in these circumstances. A minimum of two responses should be displayed to demonstrate reproducibility i.e., at least one close replication. Several recordings may be needed to establish consistency and to enable meaningful interpretation. It may be beneficial to superimpose repeated PERG recordings to evaluate quality and reproducibility.

### PERG analysis and reporting

#### Interpretation

The amplitudes of the standard PERG components are measured between the peaks and troughs of a grand average of the most consistent waveforms. The P50 amplitude is measured from the trough of N35 to the peak of P50. The N95 amplitude is measured from the peak of P50 to the trough of N95. It should be recognized that measured in this way, N95 amplitude includes the P50 amplitude and P50 that of N35. In cases where the N35 is poorly defined, the P50 amplitude is measured from the average pre-stimulus baseline (or between time zero and the onset of P50) to its peak. The N95 depends upon the preceding P50 and if P50 is reduced due to macular cone dysfunction the N95 will show proportionate reduction. The N95:P50 amplitude may help identify selective or predominant loss of N95 amplitude. Retinal ganglion cell dysfunction will reduce N95 amplitude more severely than the P50 amplitude, and there may be a reduction in the slope of the descending limb of the P50 component. Reduction in the RGC contribution to the PERG may also shorten the P50 component peak time, and in such cases P50 often shows mild-moderate reduction (maximum reduction about 70%). It is possible for P50 to be abnormally shortened, without detectable reduction in the N95:P50 amplitude e.g., if the pre-morbid N95:P50 amplitude was high, the reduced response may still fall within the reference range.

P50 peak time should be measured from the onset of the contrast reversal to the peak of the component of interest. It should be noted that the highest absolute amplitude point on a waveform will not always be appropriate for the definition of the peak if there is contamination from muscle activity or other artefacts. The peak should be designated where it would appear on a smoothed or idealized waveform (see Fig. 1), taking care to identify when P50 is genuinely abnormally shortened in peak time or delayed.

#### Reporting

It is recommended that all reports contain measurements of P50 and N95 amplitude (see above), and P50 peak time, while the N95 peak time is usually not analyzed (precise determination of peak time is often impossible due to a broader shape of N95). Measurements of P50 and N95 can be confounded by baseline drift, and this should be taken into account when measuring waveforms. Some propriety analysis software uses the averaged pre-stimulus baseline and similar time average at the end of the trace to fit the linear trend and remove the drift from the data. It should be noted if trend removal is used for measurements. The report should also include a description of any waveform abnormality, such as a shallow slope between P50 and N95.

All reports should also contain the stimulus parameters, (check width, stimulus contrast, mean luminance, field size, ambient room illumination and definition of “time zero” if it differs from the convention (see Sect. "Visual display units (VDUs) for stimulation").

Note any atypical pupil size or fixation instability and the relevant comparative reference ranges. The report of PERG results should include the recorded waveforms with appropriate amplitude and time calibrations, marks for the N35, P50 and N95 components, and should show replications.

## Reference ranges

Reference ranges for standard PERGs are specific to the type of electrode and each laboratory should use suitable reference data to interpret patient PERGs. Establishing reference values involves recruiting and testing sufficient reference subjects per partition. Pattern ERGs in adults are relatively stable up to the 5th decade, but age-specific reference limits should be considered especially for the elderly population, when other factors such as age associated neuronal loss and senile miosis, can alter the response. Subjects should be matched to the patient population in demographic factors. Reference limits should be constructed using non-parametric or robust techniques to enclose the central 95% of values, i.e., the 2.5th and 97.5th percentile.

Establishing laboratory-specific reference values is the optimal process. If reference data from elsewhere are used, for example, manufacturers’ data or published data, they must be verified as appropriate for local use with an understanding of possible limitations and how the reference limits were defined.

In longitudinal studies, subject-specific reference values may be more useful than a comparison with a reference group (cross-sectional population-based control values) to decide whether a parameter has changed by a clinically meaningful amount. Such assessments should consider the consistency of stimuli and recording methods, and inter-session variability due to electrode position, pupil diameter, age etc. Inter-ocular comparisons may also be of value if dysfunction is asymmetrical or unilateral.

## Optional ISCEV Standard methods

### Large field PERGs

For some applications, users should consider recording the PERG to a field of approximately 30°, in addition to the standard 15° field size. This larger field PERG can be used in conjunction with the standard field to allow an assessment both of central and paracentral macular function with the same check width. Further, a large field PERG may have an improved signal-to-noise ratio, which would be useful in patients with very abnormal responses, or when noise levels are high. A large field PERG may also be of value in patients who are unable to fixate precisely e.g., to exclude severe macular dysfunction as cause of visual impairment. In the absence of disease, the P50 amplitude to a 30° field is around twice the size of that to a standard 15° field, and to a large field the N95:P50 amplitude is typically slightly lower, although both parameters vary in the healthy population and it is essential to use an appropriate stimulus-specific reference range.

### Simultaneous PERG and PVEP

For simultaneous PERG and PVEP recordings a check size of 0.8–1.0° (48’-60’) and a reversal rate of 3 rps is specified. This check width is within the range for both the ISCEV Standard PERG (0.8° ± 0.2°) and ISCEV Standard PVEP (1° ± 0.2°). The mean of the width and the height of the stimulus field should be 15° (see Sect. "Field size and check width") or 30° (see Sect. "Large field PERGs") but must be specified. A reversal rate of 3 rps is at the lower limit for the ISCEV Standard PERG (4 rps ± 1.0 rps and is sufficiently close to the ISCEV standard PVEP (2 rps ± 0.2 rps), to enable transient PVEP recordings. By extending the acquisition time window to 250-285 ms both the PERG and PVEP can be recorded simultaneously on separate channels. Monocular, simultaneous PERG and PVEP acquisition may be helpful in cases when active defocus is suspected, patient compliance is limited or when longer test sessions with multiple tests may not be possible. Reports should explicitly state the use of simultaneous recording, the stimulus check size and reversal rate, and interpretation based on reference data to identical stimuli.

## Non-standard methods

### Non-standard temporal rates – the steady-state PERG

At reversal rates higher than 6 rps, the successive waveforms overlap and a steady-state PERG (SS-PERG) is generated. While we realize that not all instruments are suitable to record and accurately measure SS-PERGs, there are several advantages in using it whenever possible. The main advantage is that the evaluation of response amplitude and phase (latency) is objective. Other advantages are that robust averaging and isolation of the response component at the reversal rate reduces the noise and minimizes variability. Reliable SS-PERGs can be also obtained with skin electrodes.

For steady-state PERG, a reversal rate of approximately 16 rps ± 20% is recommended. Interpretation of steady-state PERGs requires measurement of amplitude and phase shift (relative to the stimulus) of the response at the reversal rate (equal to the second harmonic) using Fourier analysis. The presence of a significant fundamental (first harmonic, i.e., at 8 Hz for 16 rps) indicates technical problems. For correct interpretation, the analysis period must be an integer number of stimulus cycles; more than 6 cycles are advised for robust signal detection. Steady-state PERG recording without the capability for such analysis is not recommended.

Reporting of steady-state PERGs should include the amplitude and phase shift of the PERG at the reversal rate. If phase shift rather than peak time is reported, phase shift needs to be uniquely defined e.g., whether it is increasing or decreasing with increasing peak time. *Phase* or *phase shift ϕ* for a certain harmonic (spectral component) is typically defined as an angle in degrees such that the time course of the harmonic is given by:

$${{A(t)  =  cos (2}} \cdot \pi  \cdot {{(f}} \cdot t - {{ }}\varphi {{/36}}{{{0}}^ \circ })).$$

More useful in our domain is a specification of the corresponding delay time *t*_*ϕ*_ = (*ϕ* / 360°) / *f*:

$${{A(t)  =  cos (2}} \cdot \pi  \cdot {{f}} \cdot (t - t\varphi )),$$

where

*A(t)*: amplitude over time of the pertinent harmonic at frequency *f*

(e.g., for pattern reversal: twice the fundamental frequency);

*f*: frequency in Hertz (= 1 / seconds);

*t*: time in seconds;

*φ*: phase angle in degrees;

*t*_*φ*_: phase delay time in seconds.

This definition has the following properties:

- When *A(t)* has a peak at time zero (i.e., is a pure cosine), phase *ϕ* and phase delay time *t*_*ϕ*_ are zero.
- A delayed response corresponds to positive phase delay-time values.

### Non-standard large check widths

Additional recordings to a coarse stimulus e.g., only 4 squares within the stimulus field contain more luminance components than standard responses. However, responses to such stimuli may provide a useful control in the presence of significant uncorrected refractive error or mild media opacity, as likely to be less attenuated than the response to finer or standard patterns, informing interpretation of the ISCEV Standard PERG.

## Abbreviations

ISCEV: International society for clinical electrophysiology of vision
PERG: Pattern electroretinogram
PVEP: Pattern reversal visual evoked potential
Rps: Reversals per second
Hz: Hertz
mfERG: Multifocal ERG

## Declarations

### Statement on the welfare of animals

Preparation of this Standard did not involve research using animals.
